# What is measured and what is estimated in body composition? A clarification framework for terminology and interpretation

**DOI:** 10.1007/s11154-026-10071-4

**Published:** 2026-07-03

**Authors:** Francesco Campa, Henry C. Lukaski, Tatiana Moro, Grant M. Tinsley, Antonio Paoli, Giuseppe Coratella

**Affiliations:** 1 Department of Biomedical Sciences, University of Padua, Padua, Italy; 2 Department of Kinesiology and Public Health Education, University of North Dakota, Grand Forks, USA; 3 Energy Balance & Body Composition Laboratory, Department of Kinesiology & Sport Management, Texas Tech University, Lubbock, TX USA; 4 Department of Biomedical Sciences for Health, Università degli Studi di Milano, Milan, Italy

**Keywords:** Anthropometry, Body composition assessment, Bioelectrical impedance analysis, Methodology, Nutritional assessment

## Abstract

Body composition assessment has traditionally focused on estimating body mass components and the estimation processes required to obtain them in vivo. Within this context, the five-level model and the methodological taxonomy proposed by Wang and colleagues provided a rigorous conceptual framework that has strongly shaped the field. However, contemporary research and practice reveal persistent ambiguity in the use of terms such as direct, indirect, and doubly indirect. This ambiguity has become increasingly evident as methods such as anthropometry and bioelectrical impedance analysis are widely used not only to estimate body mass components, but also to obtain whole-body variables that are directly measured and interpreted as clinically, physiologically, or performance-relevant descriptors. Confusion often arises when directly measured outputs (e.g., skinfolds, girths, phase angle) are not clearly distinguished from model-derived estimates of body composition (e.g., fat mass, fat-free mass). Accordingly, we propose a clarification framework for in vivo body composition assessment that distinguishes: (1) directly measured whole-body variables, (2) model-derived estimates of body composition, and (3) hybrid assessment pathways combining measured and inferred outputs. In this framework, classification depends on the specific variable of interest and the estimation process linking measurement to output, rather than on assigning a fixed label to the instrument itself. The term direct refers only to an output that is measured rather than model-derived, and does not imply direct assessment of body components, greater validity, or superior methodological accuracy. The proposed framework provides a practical conceptual tool for distinguishing measured from estimated body composition outputs, thereby improving methodological transparency, interpretation, and reporting consistency across research and clinical practice.

## Body composition: conceptual framework and variables

Body composition research has traditionally focused on the methods and in vivo estimates of body mass components in steady state or dynamic conditions [1]. Wang and colleagues [2] proposed a five-level hierarchical model of biological organization, ranging from the atomic to the whole-body level, that notably advanced body composition research. This framework provided a coherent biological structure for organizing body composition-related variables and for understanding how body mass may be conceptually partitioned across levels of increasing complexity. Within this framework, the core domain of body composition is represented by body mass components, which are organized across the atomic, molecular, cellular, and tissue–organ levels (levels I–IV) [2, 3]. These include, among others, fat mass, fat-free mass, total body water and its compartments, bone mineral content, adipose tissue, and skeletal muscle mass. Because these components are not directly measurable in vivo, their assessment necessarily relies on inferential procedures grounded in physical, chemical, physiological, or statistical models [1].

**Fig. 1:**
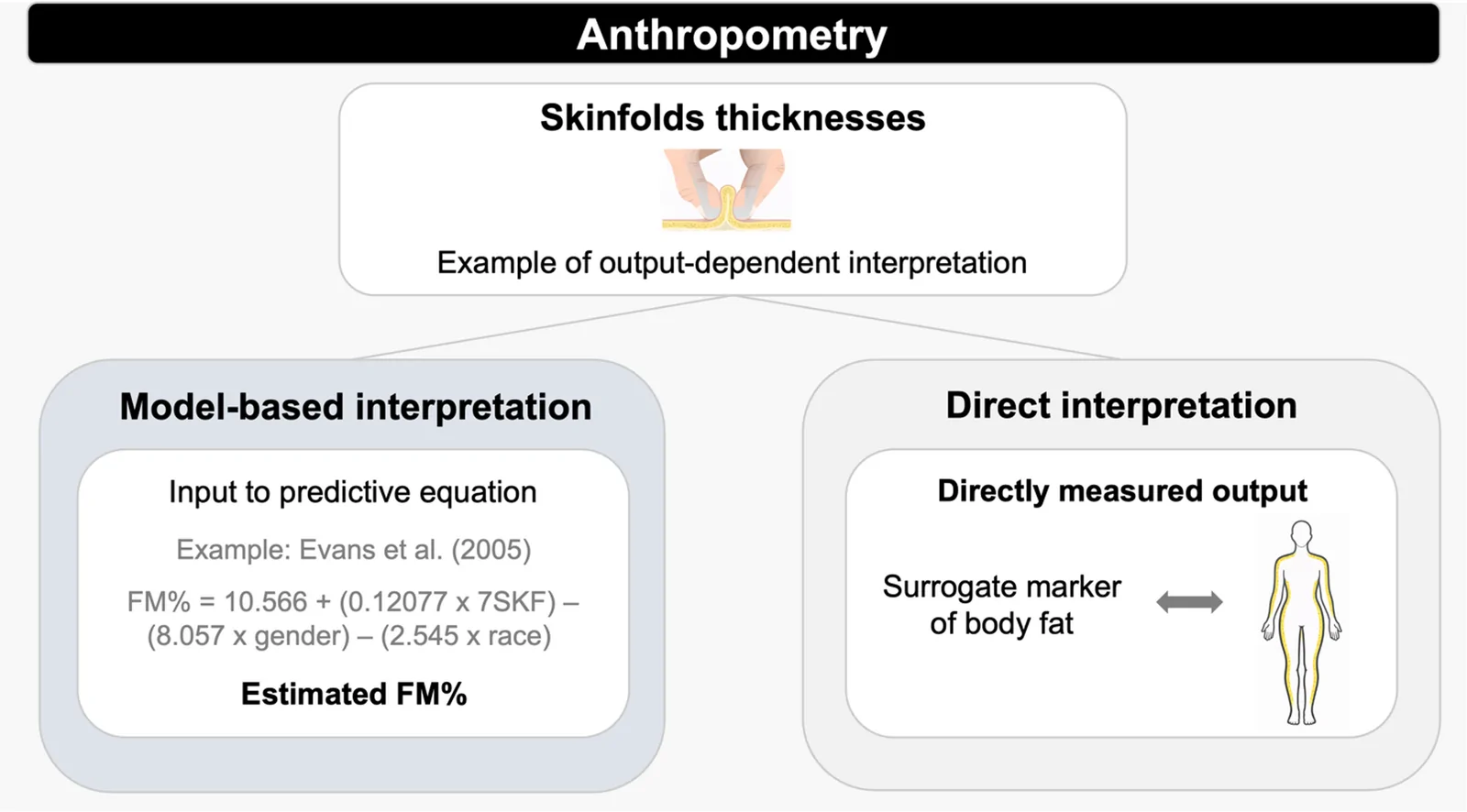
Example of output-dependent interpretation in anthropometry. Skinfold thicknesses may be interpreted directly as surrogate markers of adiposity or used within predictive equations (e.g., Evans et al., [13]) to estimate percentage fat mass (FM%). This example illustrates how the same measured variable may serve different roles depending on its analytical use. Notes: 7SKF = sum of seven skinfolds (subscapular, triceps, chest, midaxillary, iliac crest, abdominal, and thigh); gender: 0 = female, 1 = male; race: 0 = White, 1 = Black

Contemporary research and applied practice increasingly rely on whole-body measurements associated with body composition (i.e., those related to the size, shape, and exterior or physical characteristics of the body), even when they do not represent body mass components themselves [4]. These variables include anthropometric dimensions and indices, skinfold thicknesses, girths, body mass, body volume, body density, and bioelectrical variables such as resistance, reactance, phase angle, and vector patterns derived from bioelectrical impedance analysis (BIA) [5, 6]. These variables also include externally derived variables obtained through digital imaging approaches, such as two-dimensional and three-dimensional optical imaging, which provide body shape descriptors and volumetric estimates increasingly used in body composition research and practice [7]. Such outputs are often used as clinically, physiologically, or performance-relevant descriptors in their own right, or as surrogate markers of malnutrition risk, prognosis, hydration status, soft tissue condition, or related body composition phenotypes [8, 9]. This distinction is conceptually important, as a directly measured whole-body variable is not equivalent to a directly measured body component. For example, a skinfold thickness, a waist girth, or a phase angle may provide meaningful information related to body composition [10], but none of these constitutes a direct measurement of an internal mass-based component such as fat mass or fat-free mass. Likewise, the same variable may function differently depending on context, serving either as a descriptive marker or as an input for estimating body mass components [11, 12]. For example, the sum of skinfolds may be reported directly as an indicator of subcutaneous adiposity, or it may be entered into a predictive equation to estimate fat mass or percentage fat mass, as shown in Fig. 1.

**Fig. 2:**
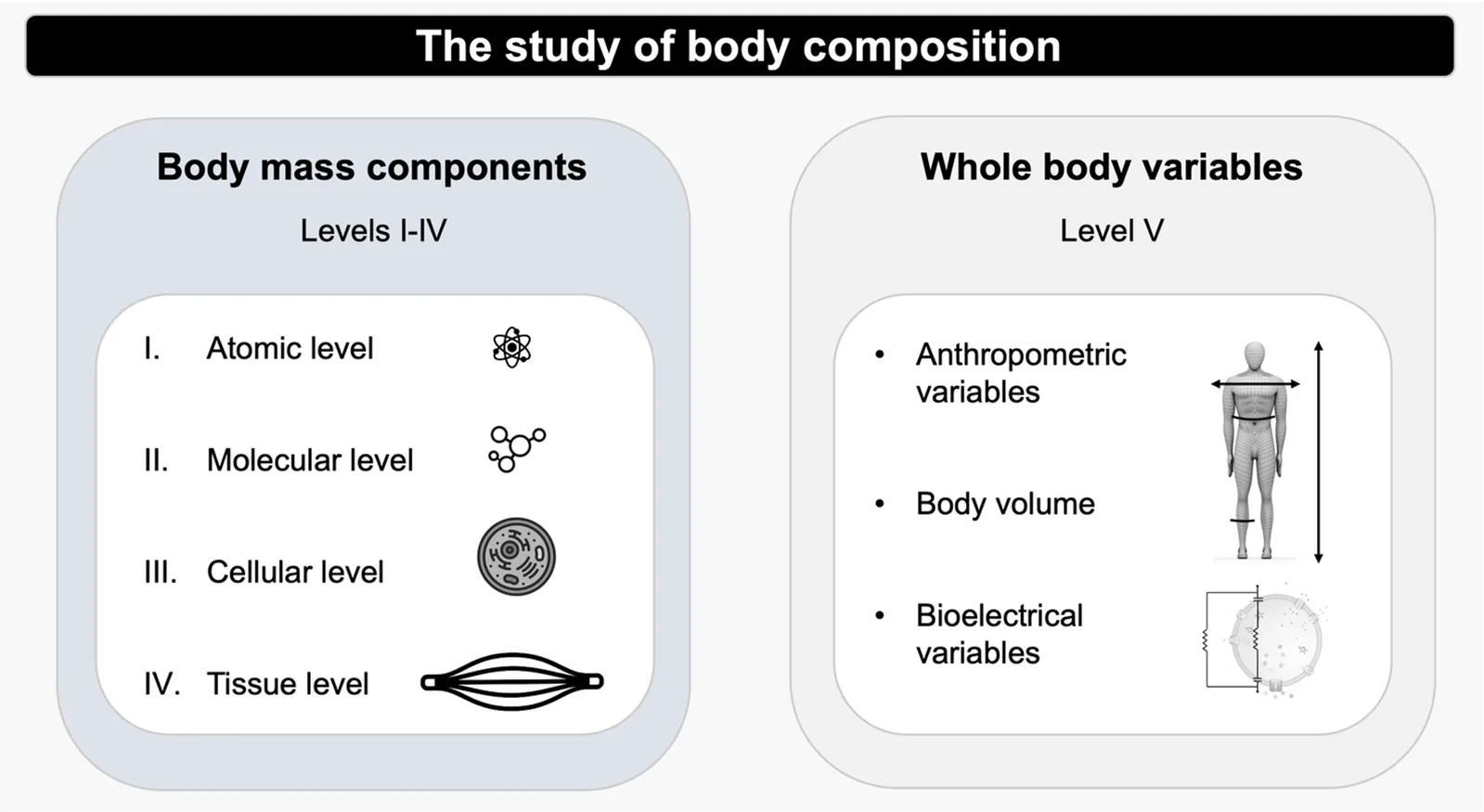
Conceptual distinction between body mass components and whole-body variables related to body composition. Body mass components are organized across the atomic, molecular, cellular, and tissue–organ levels (Levels I–IV) and represent the material constituents of body mass. Whole-body variables, such as anthropometric and bioelectrical measures, are directly measurable properties that are related to body composition but do not constitute body mass components per se. These variables provide descriptive information, serve as markers, or act as inputs for inferential models used to estimate body components

Similarly, resistance and reactance may be interpreted directly through phase angle or vector analysis, or they may be used within prediction models to estimate total body water or fat-free mass. The conceptual separation between body mass components and whole-body variables is illustrated in Fig. 2.

Body composition methodology has primarily been organized around the estimation of body mass components [3], reflecting both the scientific priorities of the field and the available assessment technologies [14]. Wang et al. [15] proposed a rigorous classification system for methods used to determine body mass components, linking measurable quantities to inferred components through statistical or theory-based functions. This contribution remains highly valuable and conceptually robust [16, 17]. However, it was developed in a context where whole-body variables were primarily considered intermediate measurable quantities rather than outputs of interest in their own right [18]. In contemporary research the use of whole-body variables as standalone outcomes has expanded substantially [5, 19], and this trend is particularly evident in sport and exercise science, clinical monitoring, and field-based applications [8, 19–21]. Indeed, whole-body outputs are often selected not because they estimate a body component, but because they are feasible, repeatable, and informative in applied settings [22]. This distinction may be especially relevant in clinical nutrition and related healthcare settings, where directly measured variables may have practical value independently of compartment estimation. For example, anthropometric measures such as mid-upper arm girth may help identify malnutrition risk or muscle depletion when interpreted directly, although they are also commonly used as independent variables in regression-based models to estimate skeletal muscle mass [23]. Similarly, directly measured bioelectrical variables (i.e., resistance, reactance, phase angle, vector length) may provide clinically meaningful information on nutritional status or sarcopenia-related risk without requiring conversion into estimated body composition compartments [24, 25]. In these contexts, the value of the measurement lies not in its ability to directly indicate a component, but in its role as a clinically interpretable marker [26]. As a result, a growing conceptual ambiguity has emerged in the terminology used to classify body composition assessment methods. In particular, labels such as direct, indirect, and doubly indirect are frequently applied inconsistently and often without explicit reference to the variable or output of interest [11, 27], creating a recurrent source of confusion. When this distinction between a directly measurable variable and an estimated variable is not made explicit, methodological labels risk obscuring rather than clarifying the actual assessment process. This evolution also includes the emergence of digital imaging techniques, which have expanded the range of quantifiable whole-body variables and introduced new analytical approaches for deriving body composition-related outcomes [28].

The present review proposes a clarification framework for in vivo body composition assessment based on the nature of the output and the estimation process linking measured variables to the output. Rather than assigning a fixed methodological label to an instrument or technique, the proposed framework distinguishes between: (1) directly measured whole-body variables, (2) model-derived estimates of body composition, and (3) hybrid assessment pathways combining measured and inferred outputs. In this context, the term direct refers only to the output that is measured rather than model-derived, and does not imply direct assessment of body components, greater validity, or superior methodological accuracy. By making these distinctions explicit, the proposed framework aims to improve conceptual clarity, methodological transparency, and consistency in the interpretation and reporting of body composition assessment across clinical, research, and applied settings. The goal is to provide a clearer output-based framework that preserves the inferential logic of body composition assessment while making explicit whether a given result corresponds to a directly measured whole-body variable, a model-derived estimate, or a hybrid analytical output. The present article is a conceptual and methodological analysis aimed at clarifying the terminology used in body composition assessment. The framework was developed through critical examination of existing body composition models, methodological taxonomies, and contemporary applications of anthropometry, bioelectrical impedance analysis, imaging, and multicomponent assessment methods.

## Methodological classification in body composition assessment: Wang’s framework and its contemporary interpretation

**Fig. 3:**
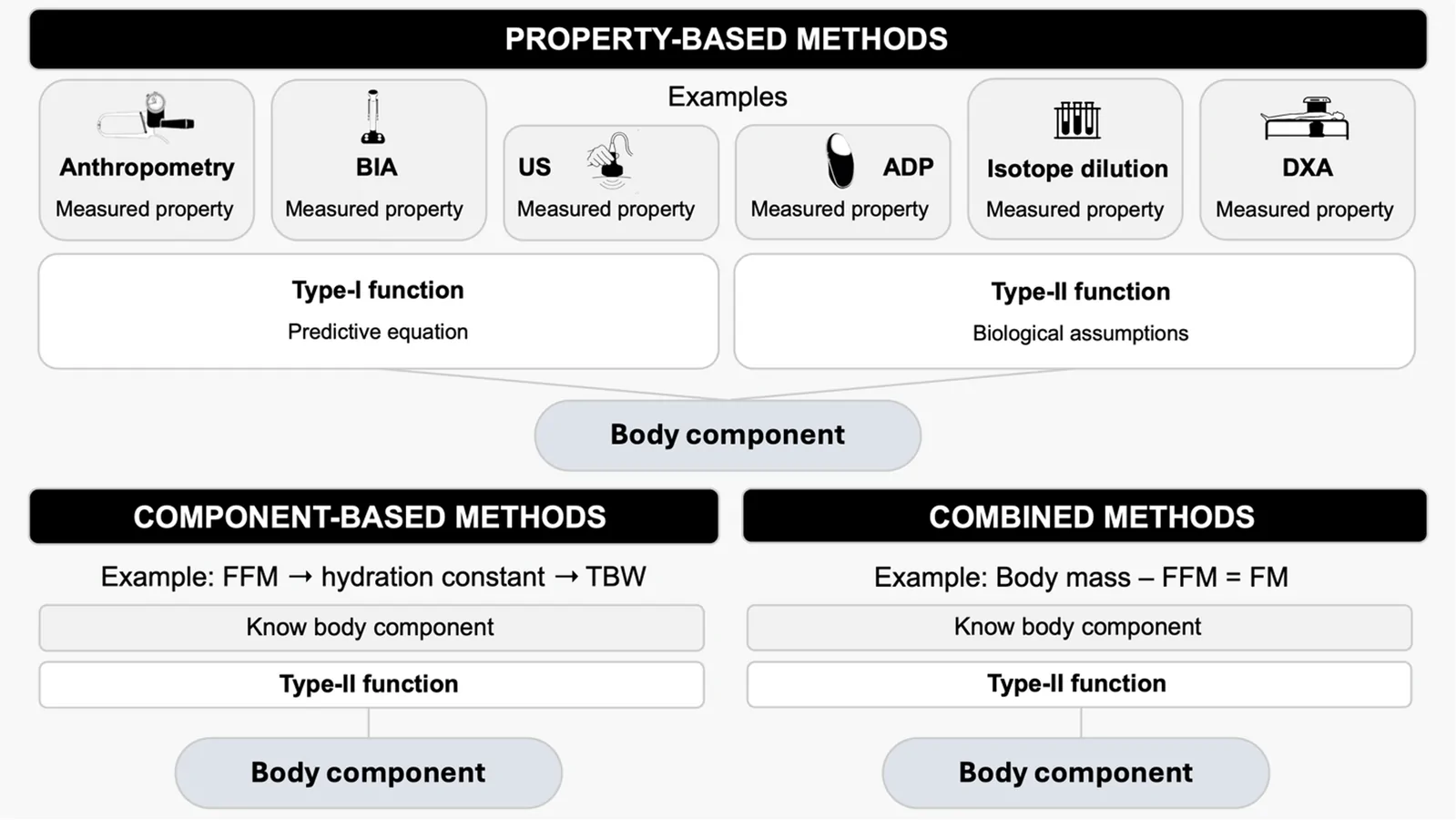
Conceptual pathways linking measurable quantities to body components in body composition assessment [15]. Property-based methods derive body component outputs from directly measurable physical, dimensional, or electrical quantities through either Type-I functions (typically implemented through prediction models) or Type-II functions based on biological or physical assumptions. Component-based methods derive one body component from another previously known or estimated component, whereas combined methods integrate multiple measurable quantities and/or component estimates within the same analytical framework. This conceptual structure illustrates how different inferential pathways may lead to body composition outputs. ADP, air displacement plethysmography; BIA, bioelectrical impedance analysis; CT, computed tomography; DXA, dual-energy X-ray absorptiometry; US, ultrasound; TBW, total body water; FFM, fat-free mass

In the Wang’s methodological nomenclature [15], methods are classified according to the nature of the measurable quantity (e.g., property-based, component-based, or combined) and the type of inferential function used to derive the body composition estimates. Two broad categories of inferential functions were identified. Type-I functions correspond to empirical or statistical relationships, typically derived through regression models calibrated against reference data. These are commonly used when body components are predicted from measurable quantities such as anthropometric, bioelectrical, or ultrasound-derived variables. Type-II functions, by contrast, are grounded in mechanistic or theory-driven relationships based on physical, physiological, or biological principles. These include, for example, densitometric approaches based on Archimedes’ principle, isotope dilution methods for estimating total body water, and attenuation-based models used in dual-energy X-ray absorptiometry (DXA) [29]. In some cases, body components may be estimated through a hybrid approach that integrates biological assumptions (Type-II function) with predictive equations (Type-I function). Nonetheless, the distinction between Type-I and Type-II functions remains highly valuable because it clarifies that the estimation of body mass components necessarily depends on inferential pathways rather than on direct access to the component itself. In this sense, the Wang’s model [15] provides a rigorous conceptual bridge between measurable outputs and the biological components of interest. It also helps explain why different techniques may produce estimates of the same body component through distinct inferential routes. Figure 3 illustrates this working model. Property-based approaches use measurable physical, dimensional, or bioelectrical variables as inputs to estimate body mass components. Component-based approaches derive one component from another already known or previously estimated. Combined approaches integrate multiple measurable quantities and/or component estimates within the same analytical framework. Taken together, these categories provided one of the first systematic attempts to organize body composition methodology according to the structure of the inferential process rather than the instrument alone.

Despite its enduring relevance, the Wang’s classification was developed in a period in which the main scientific and clinical emphases were on the estimation of body mass components [15]. Thus, the framework was not intended to provide an explicit operational classification for the growing number of whole-body variables that are now frequently measured and interpreted as outcomes. This is particularly evident in contemporary applications of anthropometry, BIA, ultrasound, and related techniques, which are often used not only to estimate components such as fat mass, fat-free mass, or skeletal muscle mass, but also to quantify and monitor variables such as skinfold thicknesses, girths, phase angle, or muscle thickness [8, 9, 20]. This shift does not diminish the value of the Wang’s framework; rather, it highlights the need for a complementary clarification. Specifically, there is now a greater need to distinguish between: (1) variables that are directly measured, (2) body composition variables that are inferred from those parameters, and (3) analytical pathways that combine measured and modelled outputs. Without such a distinction, methodological labels like direct, indirect, and doubly indirect may be applied inconsistently and may fail to discriminate between what is actually measured or estimated in each assessment context. Therefore, the present paper does not seek to replace the Wang’s taxonomy, but to extend its interpretive usefulness considering contemporary research practice.

## An output-based framework for body composition assessment

Although the Wang classification [15] remains conceptually rigorous for organizing inferential pathways used to estimate body mass components, contemporary body composition research increasingly involves outputs that do not fit neatly into a component-centred classification alone. Many techniques currently used in clinical, research, and applied settings generate outputs that are directly measured and interpreted as meaningful descriptors or markers, even when they are not used to estimate body mass components per se [27, 30]. This scenario has contributed to a growing ambiguity in the terminology used to classify body composition assessment methods. Labels such as direct, indirect, and doubly indirect are often applied inconsistently across the literature and frequently without explicit reference to the variable or output of interest [27, 30–33]. The same technique may be described differently depending on whether attention is focused on the instrument, the measurement process, or the inferred biological interpretation. When these distinct outputs are not explicitly differentiated, methodological labels may become misleading and may obscure rather than clarify the actual assessment pathway. The present framework proposes that body composition assessment should be organized primarily according to the nature of the output and the inferential pathway required to obtain it. Specifically, the framework distinguishes between:

1. directly measured whole-body variables,
2. model-derived estimates of body composition,
3. hybrid assessment pathways that combine measured and model-derived estimates within the same analytical process.

Importantly, the proposed classification should not be interpreted as a hierarchy of methodological quality. Direct outputs are not inherently more valid, accurate, or clinically useful than model-derived estimates. Rather, the framework only distinguishes whether interpretation relies on measurement alone or on additional inferential procedures. Within this updated approach, classification is not treated as a fixed property of an instrument or technique, but as a function of the specific output being generated. The same technique may therefore occupy different positions within the framework depending on whether it is used to provide a measured variable, a model-derived estimate, or a hybrid analytical result. Representative examples of the proposed framework are summarized in Table 1.

**Table 1:** Output-based classification framework for in vivo body composition assessment

Main category	Subtype	Description	Key characteristics	Representative outputs / Examples
1. Directly measured whole-body variables	—	Outputs obtained through direct measurement procedures or simple arithmetic derivation from primary measured quantities.	• Output is measured • Standardized procedures • ≠ body mass components	• Anthropometric dimensions • Derived anthropometric descriptors: BMI, WHR, sum of skinfolds • Raw bioelectrical variables • BIVA descriptors
2. Model-derived estimates of body composition	2a. Mechanistic (theory-driven) estimates	Outputs derived from measurable quantities through physical, physiological, or biological principles.	• Based on explicit theory-driven assumptions • Output is inferential	• TBW from isotope dilution • FM from densitometry • Bone mineral content from DXA • Body volume from theory-based DXA models or 3DO and 2DO systems
	2b. Empirical (data-driven) estimates	Outputs corresponding to body mass components derived through prediction models.	• Model-dependent • Population-specific • Output is inferential	• FM, FFM, TBW, or SMM from anthropometric, BIA, 3DO, 2DO or ultrasound-based equations
3. Hybrid assessment pathways	—	Analytical pathways combining directly measured outputs and model-derived estimates.	• Integrates measured and inferred quantities • Common in multicomponent and applied models	• Conventional 4 C model • Rapid/fast 4 C model • FM derived as body mass minus estimated FFM

*3DO* 3-dimensional optical imaging, *2DO* 2-dimensional optical imaging, *4C* four component, *ADP* air displacement plethysmography, *BIA* bioelectrical impedance analysis, *BIVA* bioelectrical impedance vector analysis, *BMI* body mass index, *DXA* dual-energy X-ray absorptiometry, *FM* fat mass, *FFM* fat-free mass, *SMM* skeletal muscle mass, *TBW* total body water, *WHR* waist-to-hip ratio

## Practical applications of the proposed framework

The practical usefulness of the proposed framework becomes evident when applied to commonly used field methods. Anthropometry and BIA provide illustrative examples of how the same method or analytical approach may generate different classes of outputs depending on the variable of interest and the inferential pathway involved. In the case of anthropometry, directly measured outputs include variables such as stature, body mass, girths, and skinfold thicknesses, as well as simple arithmetic descriptors such as body mass index, waist-to-hip ratio, or the sum of skinfolds [22]. These outputs fall within the category of directly measured whole-body variables, as they are obtained without the use of predictive models aimed at estimating body mass components (Fig. 1). However, when anthropometric variables are entered into predictive equations to estimate fat mass, fat-free mass, or skeletal muscle mass, the resulting outputs become model-derived estimates of body mass components (Fig. 1). In these cases, anthropometry no longer provides the variable of interest directly but instead serves as the input to an inferential process.

A similar logic applies to BIA. When the variable of interest corresponds to directly acquired bioelectrical properties such as resistance and reactance, or to mathematical descriptors derived exclusively from these measured quantities (e.g., phase angle and vector-based descriptors), the outputs belong to the category of directly measured whole-body variables. Although phase angle is mathematically calculated from resistance and reactance, it does not require predictive equations or physiological compartment models. For this reason, within the proposed framework it is classified as a direct output derived from measured quantities rather than as a model-derived body composition estimate. These variables may be interpreted descriptively or used as markers of hydration status, soft tissue condition, or body composition-related phenotypes [9, 34], providing physical measurements that enable qualitative interpretations of physiological conditions [35, 36]. This distinction is especially relevant given the known limitations of predictive equations, which are generally valid only in populations similar to those in which they were developed [37]. In addition, some equations rely on categorical variables, such as race or ethnicity, that may be difficult to define or apply meaningfully in contemporary practice [38, 39]. This issue may be further illustrated by conventional 50 kHz phase-sensitive BIA [35]. In this context, resistance is directly measured as a physical property of the body, but it is often subsequently entered into predictive equations to estimate total body water, which may then be further converted into fat-free mass through additional physiological assumptions (e.g., a fixed hydration constant) [40]. Thus, what begins as a directly measured variable may become part of a multi-step estimation process. Each step introduces its own assumptions and sources of uncertainty, including technical measurement error and prediction error, thereby increasing the risk of propagated inaccuracy in the final estimate [41]. Beyond prediction error, all body composition assessments are affected by measurement uncertainty arising from technical, biological, and operator-related sources. Importantly, uncertainty is not limited to model-derived estimates but also applies to directly measured variables. Therefore, the distinction proposed in the present framework concerns the inferential status of the output (measured versus estimated) rather than its precision, accuracy, or overall measurement quality. One last point regards also the bioelectrical assessment. In the literature, terms such as indicator, surrogate measure, and biomarker are often used interchangeably when referring to bioelectrical outputs. However, these terms may differ depending on the intended biological or clinical interpretation. For example, phase angle may be generally considered as a variable for descriptive purposes, or as a biomarker in relation to specific health-related characteristics (e.g., inflammation, fatigue), or as a surrogate measure if associated with body mass components (e.g., intracellular-to-extracellular water ratio). Within the present framework, such outputs remain directly measured variables (or mathematical descriptors derived from measured variables), irrespective of their subsequent interpretation as physiological or clinical indicators. Consequently, all these terms are not synonyms but should be used within the appropriate context.

Imaging-based approaches provide an additional example of how the same technique may generate different types of outputs within the proposed framework. Three-dimensional optical imaging, for instance, can be used to derive whole-body variables such as body volume or surface geometry through theory-driven procedures, which fall within the category of model-derived estimates based on mechanistic principles [42, 43]. However, these same variables may also be incorporated as independent variables in regression-based prediction models to estimate body composition components [44–46]. Accordingly, three-dimensional optical imaging may contribute to both mechanistic (theory-driven) and empirical (data-driven) categories, depending on how the derived variables are used within the estimation process. Similarly, two-dimensional digital imaging techniques, such as single lateral standing imaging, provide externally derived body shape variables from planar images [7, 45]. As with three-dimensional optical imaging, these variables may be interpreted descriptively or used as independent variables in regression-based prediction models, and therefore their classification depends on the analytical use of the output rather than on the technique itself.

**Fig. 4:**
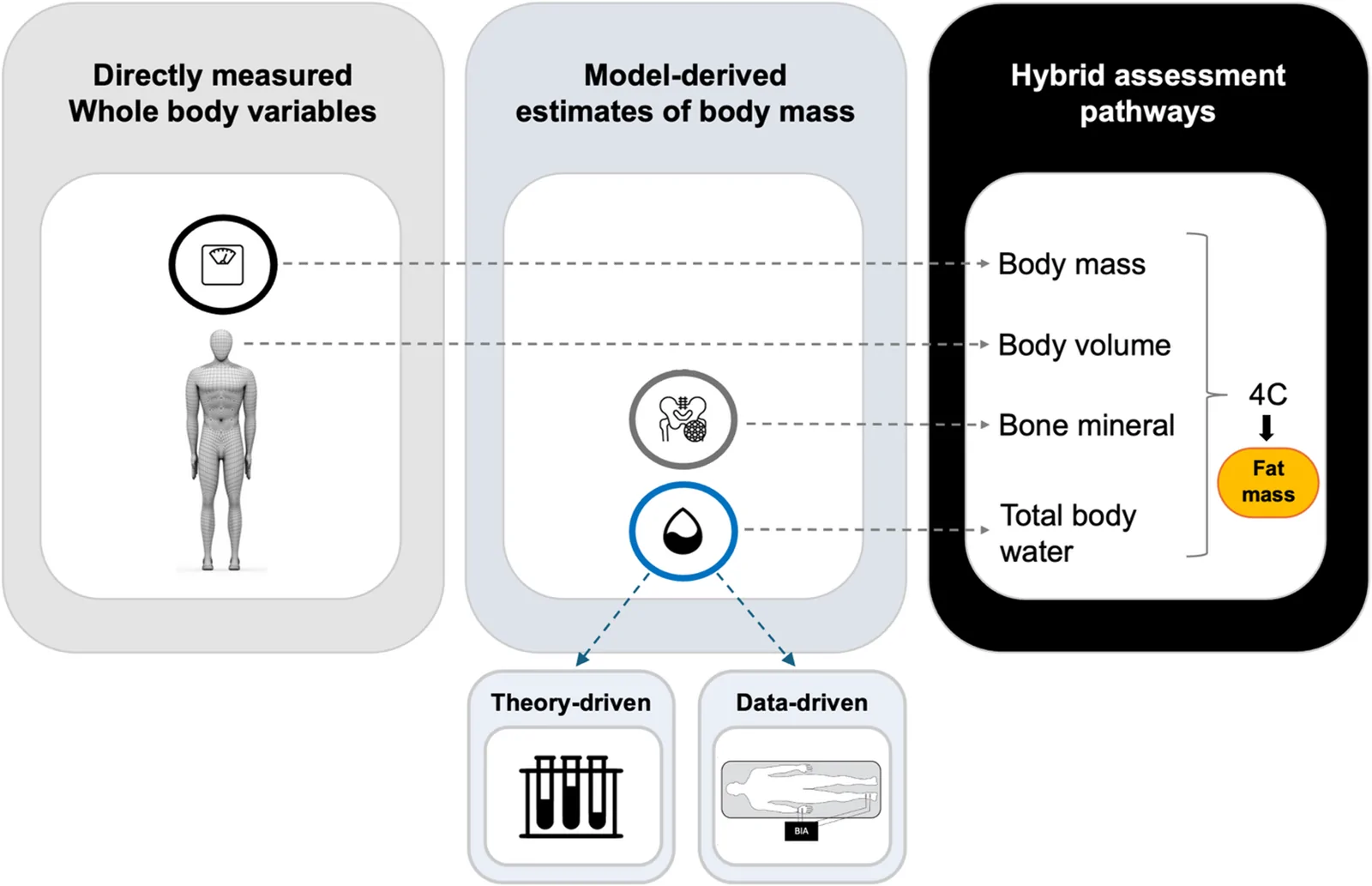
Hybrid four-component (4C) model for fat mass estimation: directly measured whole-body variables (body mass and volume) are combined with model-derived components (bone mineral and total body water) to derive fat mass

Anthropometry and BIA may contribute to hybrid assessment pathways when directly measured outputs are combined with inferred quantities within broader analytical workflows [47, 48]. Figure 4 illustrates a hybrid pathway for estimating fat mass using a four-component (4 C) model, as defined within the hybrid assessment pathways category (Table 1). In this framework, directly measured whole-body variables (body mass and body volume) are combined with model-derived components (bone mineral and total body water). Total body water can be estimated either via BIA, using data-driven procedures [12], or through dilution techniques based on a theory-driven approach [37]. These components are then integrated into a predictive equation, such as the 4 C model proposed by Wang et al. [49] (fat mass = 2.747·body volume − 0.714·total body water + 1.146·bone mineral − 2.050·body mass), highlighting the hybrid nature of the method. Body volume can also be quantified using DXA through both data-driven and theory-driven procedures [48, 50]. Overall, this example shows that the same parameter may be obtained through different pathways depending on the methodological approach.

## Summary and conclusions

Body composition assessment encompasses both measurable outputs and inferential procedures. While Wang’s conceptual and methodological frameworks remain foundational for understanding how body mass components are organized and estimated in vivo, contemporary practice increasingly relies on whole-body variables that are directly measured and interpreted as clinically, physiologically, or performance-relevant outcomes.

The present paper proposes a clarification framework that distinguishes between (i) directly measured whole-body variables, (ii) model-derived estimates of body composition, and (iii) hybrid assessment pathways that combine measured and inferred outputs. Within this framework, classification depends on the specific output being generated and the inferential pathway required to obtain it, rather than on assigning a fixed methodological label to the instrument itself. Consequently, the same technique may occupy different positions within the framework depending on whether it is used to provide a measured variable, a model-derived estimate, or a hybrid analytical result.

This distinction is not merely semantic. Failure to differentiate measured outputs from inferred estimates may contribute to misinterpretation of body composition findings, particularly when comparing populations, monitoring longitudinal changes, or evaluating secular trends in obesity, adiposity, and muscle mass. In such contexts, observed differences may reflect not only biological variation but also the assumptions embedded within predictive models and assessment methodologies. Importantly, the term direct, as used in the present framework, refers only to whether an output is measured rather than model-derived, and it does not imply direct access to body mass components, greater validity, or superior methodological accuracy. Likewise, indirect should be interpreted only in relation to the inferential status of a given output.

Overall, the proposed framework is intended as a practical conceptual tool to improve conceptual clarity, methodological transparency, and reporting consistency in body composition research and practice. By explicitly distinguishing what is measured from what is estimated, it may facilitate more accurate interpretation and communication of body composition outcomes across scientific, clinical, and applied settings. 

## Data Availability

No datasets were generated or analysed during the current study.

## Abbreviations

ADP: Air displacement plethysmography
BIA: Bioelectrical impedance analysis
BIVA: Bioelectrical impedance vector analysis
BMI: Body mass index
CT: Computed tomography
DXA: Dual-energy X-ray absorptiometry
FM: Fat mass
FFM: Fat-free mass
SMM: Skeletal muscle mass
TBW: Total body water
3DO: Three-dimensional optical imaging
2D: Two-dimensional
4C: Four-component model
